# *Pistacia atlantica* Desf. A review of its traditional uses, phytochemicals and pharmacology

**DOI:** 10.25122/jml-2017-0055

**Published:** 2018

**Authors:** Fatemeh Mahjoub, Kambiz Akhavan Rezayat, Mahdi Yousefi, Masoud Mohebbi, Roshanak Salari

**Affiliations:** 1. MD, Ph.D candidate, Department of Persian medicine, School of Persian and complementary medicine, Mashhad University of Medical Sciences, Mashhad, Iran; 2. Assistant Professor, MD, Department of Internal Medicine, School of Medicine, Mashhad University of Medical Sciences, Mashhad, Iran; 3. Assistant Professor, MD, Ph.D, Department of Persian Medicine, School of Persian and Complementary Medicine, Mashhad University of Medical Sciences, Mashhad, Iran; 4. Assistant Professor, MD, Endocrine research center, Mashhad University of Medical Sciences, Mashhad, Iran; 5. Assistant Professor of Drug Control, Ph.D, Department of Pharmaceutical Sciences in Persian Medicine, School of Persian and complementary medicine, Mashhad University of Medical Sciences, Mashhad, Iran

**Keywords:** *Pistacia atlantica*, wild pistachio, traditional medicine, Persia

## Abstract

*Pistacia atlantica* is the main herbal medicine that has been widely used in the Middle Eastern and Mediterranean areas since ancient time. *Pistacia atlantica* has been used for multiple purposes like stomach diseases, renal disorders, wounds and coughs. The aim of this study is to review its botanical characterization, traditional applications, photochemistry effects and pharmacological activities. Data in this review article was gathered from credible pharmacopeias, electronic databases such as Web of Science, Science Direct, PubMed, EMBASE, Scopus, EBSCO, Google Scholar, SID and Iran Medex and textbooks of Persian medicine such as *Canon of medicine* (*Ibn-e Sina*, 980-1037 AD) and *Makhzan-al-Advia* (*Aghili*, 18th century). The keywords were searched in Persian and English books on medicinal plants and traditional medicine. The results showed that *P. atlantica* has many medicinal properties such as antioxidant, antidiabetic, antihyperlipidemic, along with others. It can also be effective in gastrointestinal diseases. Thus, different new drugs can be formulated based on *P. atlantica* for the management of various diseases.

## Introduction

The genus Pistacia (family of Anacardiaceae) includes over 600 species. *P. vera, P. atlantica, P. terebinthus, P. khinjuk*, and *P. lentiscus* are the most famous species of Pistacia that are widely distributed in the Mediterranean and Middle Eastern areas [1, 2]. More than 40% of the world production of Pistacia spp. is from Iran [3]. Iran’s Pistacia cultivation history (3000–4000 years) represents different culinary and traditional medicinal applications for this herb. The most economical species of *Pistacia* genus is P. atlantica (wild pistachio), found in in Iran [4]. Besides Iran, wild pistachio grows in different countries like Pakistan, Greece, Turkey, and North Africa [5]. *Cabulica*, *Kurdica*, and *Mutica* are the three subspecies of P. atlantica [6]. Various industrial and traditional uses are mentioned for the main parts of wild pistachio (resin and fruit) including in foods and medicine. Recent research investigates the wide pharmacological properties from various parts of P. atlantica, such as antimicrobial, antioxidant, antidiabetic, antitumor, and antihyperlipidemic activities. In this review, traditional uses, phytochemistry and pharmacological activities of P. atlantica are described.

## Methods

This Review has been written based on Persian and modern medical textbooks. Valid Persian medical references such as *Al-Qanun Fi al-Tibb* which is called *Canon of medicine* in Latin (*Ibn-e Sina*, 980-1037 AD), *Makhzan-al-Advia* (*Aghili*, 18th century) have been chosen. Science Direct, PubMed, Scopus, EBSCO, EMBASE, SID, IranMedex, and Google scholar databases were also searched by keywords *Pistacia atlantica*, wild pistachio, traditional medicine, and Persia, up until 2017.

## Results

### Botany

*P. atlantica* is a tree with a length of 2-5 m. The branches of the tree are grayish-white and have leaves composed of 9 to 11 leaflets. Oleoresin is secreted by the trunk featuring a yellowish-green color and a mild smell. This plant is single-sex and has 5 sepals and no petals [7].

### *Pistacia atlantica* in traditional medicine

In Persian, *P. atlantica* is called *Baneh*, in English *Mt. Atlas mastic tree*, in Arabic *Butm*, in the Canary Islands *Almacigo*, and in Turkish *Melengic*. The resin of wild pistachio called Saqez. Vanoshak is the name of tree fruits that has a green thin wrapper with a stiff shell and its marrow possesses nutritional value. *Baneh* have been mentioned as ripe fruits with delicious marrow [7,8]. Iran is one of the biggest producers and exporters of *P. atlantica*.

#### Temperament of *Pistacia atlantica* in Persian medicine

According to Persian medicinal literature, all the materials in the world exhibit four main qualities: “warmth”, “coldness”, “wetness”, and “dryness” and Mizaj (temperament) is a predominant quality (or qualities). According to the previously-mentioned idea, herbal medicines have a specific temperament. Each particular part of the P. atlantica species has different degrees of temperament. Fresh fruit is warm and dry in degree 1; dried fruit is warm and dry in degree 3; resin is warm and dry in degree 2; fresh leaves and branches are warm and dry in degrees 2 and 1, respectively [9].

#### Mode of application in traditional medicine

##### Gastrointestinal effects

The fruit and resin of *P. atlantica* have beneficial effects in upper and lower gastrointestinal disorders. The resin is a stomach tonic and it is used for dyspepsia, stomach ulcer, esophagitis and gastritis. Oleoresin is an appetizer, a laxative, and it is advantageous for anal fissures. The fruit is carminative and effective in nausea and vomiting, colic, hemorrhoid, anal fissures and intestinal worms [9-12].

##### Hepatic and splenic effects

The fruit and resin of *P. atlantica* are liver tonics and have hepatoprotective properties. They are prescribed for hepatic weakness, hepatitis and ascites. The fruit is a spleen tonic and it is prescribed for splenic stagnation [9,12].

##### Neurological effects

The resin and fruit of *P. atlantica* are nerve tonics and are useful in Bell’s palsy, stroke, tetanus, seizure, tremor and headache [9,12].

##### Heart and psychological uses

This plant has beneficial effects for palpitations and syncope; its fruit also has antidepressant properties [9].

##### Respiratory benefits

*P. atlantica* (resin & fruit) are prescribed for pneumonia and productive cough due to mucolytic properties [9,12].

##### Urogenital effects

The fruit of *P. atlantica* has been known as a kidney tonic and aphrodisiac; also, it is used for nephrolithiasis. This plant (resin & fruit) has diuretic and emmenagogue properties [9,12].

##### Dermatologic benefits

This plant is effective for wound healing, scabies, lip fissures and hair loss [9,12,13].

##### Miscellaneous

The resin is a gum tissue strengthener and useful for bone fractures and musculoskeletal disorders. The fruit has been used for back pain due to its analgesic properties [9].

### Phytochemistry of *Pistacia atlantica*

The chemical entities from different phytochemical groups were isolated and characterized in *P. atlantica* and mentioned in Table 1.

#### Terpenoids

An essential oil is one of the numerous metabolites extracted from the fruits, leaf-buds, twigs, flowers, leaves, resin, and galls of *P. atlantica* [8]. The main ingredients of the essential oils reported by hydrodistillation of the resin, leaves and fruits of P. atlantica based on GC (gas chromatography) and GC/MS (gas chromatography/mass spectrometry) is monoterpene with α-pinene (42.9%) and β-pinene (13.2%) in the resin. Terpinen-4-ol (21.7%) and elemol (20.0%) are two major ingredients in the oil of the leaves. The oil of the fruits has high amounts of oxygenated monoterpenes, with bornyl acetate (21.5%) as the predominant component [14]. Spathulenol is the main component of P. atlantica leaves [15]. Triterpenes like oleanolic acid, ursonic acid, masticadienonic acid, masticadienolic acid, morolic acid, and 3-O-acetyl-3-epiisomasticadienolic acid are detected in the resin of *P. atlantica* [5, 16].

#### Phenolic Compounds

Phenols are known for their antioxidant activities which reduce the risk of different diseases such as cancers [17]. Leaf extract contains two main chemical compounds of gallic acid and gallic acid methyl ester. Luteolin, luteolin 7-glycoside, chlorogenic acid, kampferol, naringin and naringin 7-glycoside were detected from the fruit extract [5]. Flavonoid glycosides were isolated from the aerial parts, leaves and stems of *P. atlantica*. These components were revealed as kaempferol-3-glucoside, quercetin-3-glucoside, quercetin-3-galactoside, quercetin-3-rutinoside, quercetin-3-glucoside-7-galactoside, apigenin 6,8-di-C-glucoside (vicenin 2) [18]. Among flavonoids, 3-methoxycarpachromene has an antiplasmodial activity which is isolated from the aerial parts of *P.atlantica* [19].

**Table 1:** Chemical ingredients and their structure isolated from *Pistacia atlantica Desf.*

Chemical compound	Structure	Plant parts	References
1 α-pinene	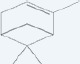	Leaf, fruit, gall, resin	[1, 14, 28, 45]
β-pinene	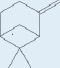	Resin	[1, 46]
limonene	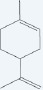	Resin, fruits	[1, 14]
Terpinolene	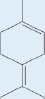	Leaf	[1, 47]
Camphene	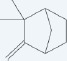	fruits	[1, 14]
Terpinen-4-ol	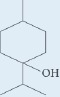	Unripe fruits	[1, 48]
Bornyl acetate	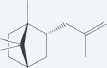	Fruits	[1, 14]
Sabinene	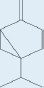	Fruits, unripe fruits	[1, 47, 48]
*p*-Mentha-1 (7),8 diene	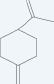	Leaf buds	[1, 48]
Δ3-carene	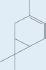	Unripe galls	[1, 49]
Spathulenol	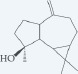	Leaf	[1, 15]
Masticadienonic acid	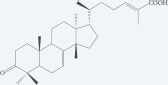	Resin	[1, 16]
Masticadienolic acid	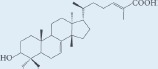	Resin	[1, 16]
Morolic acid	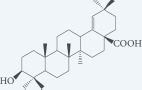	Resin	[1, 16]
Oleanolic acid	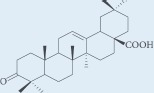	Resin	[1, 16]
Ursonic acid	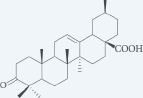	Resin	[1, 16]
3-O-acetyl-3-epiisomasticadienolic acid	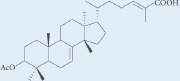	Resin	[1, 16]
Gallic acid	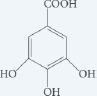	Gall and Leaf	[1, 5]
Quercetin-3-glucoside	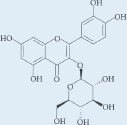	Aerial parts	[1, 18]
3-Methoxycarpachromene	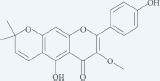	Aerial parts	[1, 19]
β-myrcene	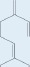	Resin, fruits	[1, 14]

#### Fatty Acids and Sterols

The fruits of P. atlantica are the main sources of unsaturated fatty acids. The ingredients of the oil content are oleic (46%), linoleic (27.5%), palmitic (24%) and stearic acid [10,20]. The main sterol of the *P. atlantica* fruit oil is β –sitosterol (87%), which is similar to peanuts and *Pistacia vera* [21]. Cholesterol, campesterol, Δ5-avenasterol, Δ7-avenasterol have been revealed from the sterol composition [10]. The sterol composition has an important role in preventing coronary heart diseases [22].

#### Miscellaneous

Tocopherols and tocotrienols are the other ingredients of *P. atlantica* hull oil with antioxidant properties [22, 23].

### Pharmacological effects

#### Antimicrobial Activities

*P. atlantica* has antibacterial properties against a large number of Gram-positive and Gram-negative bacteria. The oleoresin from *P. atlantica* var. *kurdica* with its major component -pinene has antibacterial properties against *Helicobacter pylori* [1,16]. Another research showed that acidic fractions resin of *P. atlantica* has extensively inhibitory effects against *Escherichia coli, Salmonella typhimurium, Pseudomonas aeruginosa, Alcaligenes faecalis, Enterobacter aerogenes, Pseudomonas fluorescens, Bacillus cereus, Streptococcus faecalis, Staphylococcus aureus and epidermidis* [24-27]. The methanolic extract from *P. atlantica* fresh fruits has been shown to affect fungi and yeast, such as *Candida albicans*, *Candida glabrata* and *Saccharomyces cerevisiae* [28,29]. The leaves and twigs of *P. atlantica* with its active substance 3-methoxycarpachromene showed antiprotozoal activity against *Plasmodium falciparum* [19]. In addition, *P. atlantica var. kurdica* gum could prevent cutaneous leishmaniosis from infected mice [30].

#### Antioxidant Activity

The main phenolic compounds of the fruits and leaves of *P. atlantica* are benzoic acid derivates, hydroxycinnamic acid derivative, and flavonoids that have antioxidant properties. Sinapic acid, vanillic acid and p-hydroxybenzoic acid are metabolites of hull and shell extracts with antioxidant properties [31]. A study has demonstrated the existence of new natural antioxidant ingredients isolated from the mushroom *Inonotus hispidus* growing on *P. atlantica* including methyl 5-(3,4-dihydroxyphenyl)-3-hydroxypenta-2,4-dienoate, hispolone 2 (6-(3,4-dihydroxyphenyl)-4-hydroxyhexa-3,5-dien-2-one) and hispidin 3 (6-(2-(3,4-dihydroxyphenyl)vinyl)-4-hydroxy-2H-pyran-2-one) [29, 32, 33].

#### Antihyperlipidemic effects

Research on animals revealed that P. *atlantic*a fruit oil decreased LDL cholesterol, VLDL cholesterol, triglycerides and increased HDL cholesterol. In one study, the lipid profiles were reduced in female rats with experimental hypothyroidism caused by propyl thiouracil (PTU) which received wild pistachio oil [34, 35].

#### Hypoglycemic effects

Aqueous leaf extract from *P. atlantica* has hypoglycemic effects due to the inhibitory effect on α-amylase and α-glucosidase [36, 37]. An in vivo study has also shown postprandial glucose improvement equal to glipizide and metformin and higher than acarbose in rats [37].

#### Anticancer activity

The cytotoxic effects of fruit methanolic extract from *P. atlantica* sub. *kurdica* were approved against two human cancer cell lines including the human colon carcinoma (HT29), and the human breast cancer (T47D). P. *atlantica* extract can alternate tubular protein organization with inhibitory effects on microtubule polymerization and dynamics [38-40].

#### Anticholinesterase Activity

Aqueous extracts of *P. atlantica* leaves demonstrated strong acetylcholinesterase (AChE) inhibition [41], whereas both methanol and ethyl acetate extracts of *P. atlantica* leaf exhibited relatively weak AChE inhibitory activity [42].

#### Wound-healing effects

Tanideh et al. demonstrated that the resin extract is effective in burn wounds by increasing angiogenesis, concentration of basic fibroblast growth factor (bFGF) and platelet-derived growth factor (PDGF) [43]. The results of another clinical trial showed that oleoresin of *P. atlantica* has a beneficial effect on nipple fissures and pain [44].

#### Gastrointestinal benefits

*P. atlantica* has anti-inflammatory activity and an appropriate effect in the treatment of ulcerative colitis. One study on animals exhibited that fruit oil can improve colitis in rats [43].

## Discussion

According to the Persian medical literature and recent studies, *P. atlantica* has various applications for dietetic and medicinal purposes. This review investigated extensive evidence on phytochemical and pharmacological features. Some of the therapeutic uses in traditional medicine are supported by recent studies, such as their beneficial effects on gastrointestinal disorders, but there are several pharmacological activities discussed in traditional medicine such as aphrodisiac activities, diuretic, emmenagogue, which are not confirmed by any current scientific documents, and therefore, further studies should be performed.

## Acknowledgments

This article is based on the secondary results of Dr. Fatemeh Mahjoub’s PhD postgraduate thesis.

This study was supported by the Mashhad University of Medical Sciences Research Council, Mashhad, Iran (Grant no. 950153).

## Conflict of Interest

The authors confirm that there are no conflicts of interest.
